# Impact of the epidermal growth factor receptor mutation status on the prognosis of recurrent adenocarcinoma of the lung after curative surgery

**DOI:** 10.1186/s12885-018-4849-9

**Published:** 2018-10-05

**Authors:** Tetsuya Isaka, Haruhiko Nakayama, Hiroyuki Ito, Tomoyuki Yokose, Kouzo Yamada, Munetaka Masuda

**Affiliations:** 1 Department of Thoracic Surgery, Kanagawa Cancer Center, 2-3-2 Nakao, Asahi, Yokohama, Kanagawa 241-8515 Japan; 2 Department of Surgery, Yokohama City University, 3-9 Fukuura, Kanazawa, Yokohama, Kanagawa 236-0004 Japan; 3 Department of Pathology, Kanagawa Cancer Center, 2-3-2 Nakao, Asahi, Yokohama, Kanagawa 241-8515 Japan; 4 Department of Thoracic Oncology, Kanagawa Cancer Center, 2-3-2 Nakao, Asahi, Yokohama, Kanagawa 241-8515 Japan

**Keywords:** Epidermal growth factor receptor mutation, Adenocarcinoma of the lung, Recurrence, Relapse-free survival, Post-relapse survival, Tyrosine kinase inhibitor

## Abstract

**Background:**

The prognosis of patients with epidermal growth factor receptor (*EGFR*) mutant adenocarcinoma of the lung (Mt) and *EGFR* wild-type adenocarcinoma (Wt) after complete resection of the lung differ; however, the mechanisms responsible for these differences remain unclear. The present study examined the post-operative prognosis of recurrent pulmonary adenocarcinoma patients to evaluate the clinicopathological nature of Mt and contribution of *EGFR* - tyrosine kinase inhibitors (TKI) to the prognosis of patients.

**Methods:**

The subjects were 237 patients with recurrent pulmonary adenocarcinoma who underwent *EGFR* mutation analysis, and consisted of 108 patients with recurrent Mt and 129 with recurrent Wt. Multivariate analyses were performed to investigate whether the *EGFR* status is a prognostic factor for relapse-free survival (RFS) and post-relapse survival (PRS).

**Results:**

RFS was significantly better in Mt than in Wt patients; median RFS were 20.2 and 13.3 months, respectively (*p* < 0.001). The multivariate analysis identified *EGFR* mutation as an independent prognostic factor for a favorable RFS (hazard ratio = 0.68; 95% confidence interval, 0.52–0.89). Although, no significant differences were observed in PRS between Mt and Wt patients (median PRS were 33.9 and 28.2 months, respectively; *p* = 0.360), PRS was significantly better in Mt with *EGFR* - TKI than in Wt and Mt patients without *EGFR* - TKI (*p* = 0.008 and *p* < 0.001, respectively). PRS was also significantly better in Wt than in Mt patients without *EGFR* - TKI (p < 0.001). The multivariate analysis identified the administration of *EGFR* - TKI as an independent prognostic factor for PRS (hazard ratio = 0.60; 95% confidence interval, 0.40–0.89).

**Conclusions:**

*EGFR* mutation tumors were associated with a significantly better RFS for recurrent pulmonary adenocarcinoma after curative resection of the lung, which represented the less aggressive nature of Mt tumors. However, patients with Mt did not have a favorable prognosis after recurrence unless they received *EGFR* - TKI.

## Background

The epidermal growth factor receptor (*EGFR*) mutation status has been identified as a strong predictive factor for the efficacy of *EGFR* - tyrosine kinase inhibitors (TKI). *EGFR* - TKI significantly prolong progression-free survival in patients with unresectable *EGFR* mutant adenocarcinoma of the lung (Mt) over that with chemotherapy [1–3]. Differences in clinicopathological features between Mt and *EGFR* wild-type adenocarcinoma of the lung (Wt) have recently been examined among resectable lung cancers. Radiologically, Mt has been associated with pure or mixed ground-glass opacities in computed tomography (CT) and also with a longer volume doubling time than Wt, which imply that Mt is a slow-growing tumor [4, 5]. Pathologically, Mt has been associated with a lepidic growth pattern, particularly in early stage lung cancer [6–9]. Although differences in the postoperative prognosis of patients between Mt and Wt remain controversial, most studies have demonstrated that patients with Mt have a significantly better [9–11] or slightly better prognosis than those with Wt [7, 12]. Since Mt is considered to be associated with adenocarcinoma in situ and minimally invasive adenocarcinoma, which rarely recurs after resection of the lung [9], the difference in the prognosis of Mt and Wt patients appears to strongly depend on the frequency of these low-grade adenocarcinomas.

The factors affecting the better postoperative prognosis of patients with Mt than those with Wt have not yet been identified. It currently remains unclear whether the low recurrence rate of Mt after curative surgery, slow progression after the recurrence of Mt, or therapeutic effects after recurrence, particularly *EGFR* - TKI for Mt patients, results in the better postoperative prognosis of patients with Mt In the present study, the clinicopathological features and postoperative prognosis (relapse-free survival [RFS] and post-relapse survival [PRS]) of Mt were retrospectively analyzed and compared with those of Wt.

## Methods

### Patients and follow-up

Among 1903 patients who underwent complete resection of the lung and lymph node dissection for pathological stage I-III primary lung adenocarcinoma between January 2002 and March 2016, 270 patients (14.2%) developed recurrence. Among the patients with recurrent adenocarcinoma of the lung, 237 (87.8%) underwent an *EGFR* mutation analysis and they were included in the present study. Patients who received preoperative chemotherapy or radiotherapy were excluded from this study (*n* = 70). Lobectomy was performed for the curative resection of lung cancer localized within a single lobe. Pneumonectomy was also performed if the tumor extended to multiple lobes or the central bronchus. Segmentectomy was performed for high-risk patients who were considered unable to tolerate lobectomy. Patients who underwent wedge resection of the lung were excluded from this study (*n* = 224). Curative surgery was performed without induction therapy for patients with clinical stage III if they had resectable clinical N0–1 (such as clinical T3 N1 and T4 N0–1) or clinical single-station N2 disease. Chemoradiotherapy was performed for patients with clinical multi-station N2 stage III. Systemic mediastinal lymph node dissection or sampling was performed along with resection of the lung. Staging was based on the 7th Edition of the TNM Classification for Lung and Pleural Tumors.

Patients received a chest X-ray and blood examination, including a tumor marker analysis, such as carcinoembryonic antigen and sialyl Lewis-x antigen, regularly every 3–6 months for 1–3 years after surgery and every 6–12 months for 4–5 years after surgery on an outpatient basis. CT was routinely performed 1–2 times for 1 year. Chest X-rays, blood examinations, and CT were performed when patients showed subjective symptoms. When recurrence was suspected, head magnetic resonance imaging, positron emission tomography - CT, or bone scintigraphy was additionally performed in order to identify other recurrent sites. Based on these examinations, patients were diagnosed with recurrence at a joint conference consisting of thoracic surgeons, respiratory physicians, and radiologists. Proposed treatment plans, such as whether patients need to receive *EGFR* - TKI (e.g. gefitinib, erlotinib, and afatinib), cytotoxic agents, radiation, surgery, or best supportive care, were also decided.

### Definition of terms

RFS was defined as the length of time after surgery without any sign of recurrence. New lesions considered to be metachronous multiple lung cancers were not defined as recurrence. PRS was the length of time from recurrence to the last confirmation date or date of death. RFS and PRS were examined for 237 patients with recurrent adenocarcinoma of the lung. The site of recurrence was classified into either locoregional recurrence or systemic recurrence based on initial recurrent sites. Locoregional recurrence was defined as recurrence in the ipsilateral lung, pulmonary hilum, or mediastinal, neck, axillary, or supraclavicular lymph nodes. Systemic recurrence was defined as recurrence other than locoregional recurrence; systemic recurrence included recurrence in the contralateral lung, brain, liver, adrenals, and bone, and pleura dissemination.

### EGFR mutation analysis

DNA was extracted from formalin-fixed paraffin-embedded lung cancer tissue from surgical specimens. The fragment method was performed to detect the *EGFR* exon 19 deletion mutation, and the Cycleave method was conducted to detect the *EGFR* exon 18 mutation (G719X), *EGFR* exon 20 mutation (T790 M), and *EGFR* exon 21 mutation (L858R and L861Q) [13]. A loop-hybrid mobility shift assay (LH-MSA) was also used to detect the above-described *EGFR* mutations [14].

### Statistical analysis

The clinicopathological backgrounds of Wt and Mt patients were compared using the Student’s *t*-test for continuous variables and Fisher’s exact tests for categorical variables. RFS and PRS for Wt and Mt patients were analyzed by the Kaplan-Meier method and compared by Log-rank tests. Multivariable analyses for RFS and PRS were performed using Cox’s proportional hazard regression model. A *P* value< 0.05 was considered to be significant.

## Results

The mean age of all 237 patients was 66.3 (38–86) years, and 133 patients (56.1%) were male. Lobectomy was performed on 228 patients (96.2%) (Table 1). The mean observation periods after surgery and relapse were 48.9 (4.2–132.5) months and 25.2 (0–115.3) months, respectively. Systemic recurrence was the common recurrent pattern among all recurrent adenocarcinomas of the lung (165 patients, 69.6%). Among 115 patients with pathological stage III, clinical N0–1 was observed in 97 patients (84.3%) and incidental pathological N2 in 86 (74.8%). Mt was observed in 108 patients (45.5%), and among them, mutations in *EGFR* exons 18, 19, 20, and 21 were observed in 5 (2.1%), 56 (23.6%), 1 (0.4%), and 46 patients (19.4%), respectively. There were 129 patients (54.4%) with Wt.

**Table 1:** Clinicopathological features of patients with recurrent adenocarcinoma of the lung

Total *n* = 237	
Mean age, year (range)	66.3 (38–86)
Male, (%)	133 (56.1%)
Surgical procedure, (%)	
pneumonectomy	5 (2.1%)
lobectomy	228 (96.2%)
segmentectomy	4 (1.7%)
Pathological stage, (%)	
I	60 (25.3%)
II	62 (26.2%)
III	115 (48.5%)
Recurrence pattern, (%)	
locoregional	72 (30.4%)
systemic	165 (69.6%)
*EGFR* status, (%)	
mutant	108 (45.6%)
exon 18	5 (2.1%)
exon 19	56 (23.6%)
exon 20	1 (0.4%)
exon 21	46 (19.4%)
wild-type	129 (54.4%)

*EGFR*, epidermal growth factor receptor

The clinicopathological backgrounds of Wt and Mt patients were compared in Table 2. Mt was more common in females (*p* < 0.001) and non-smokers (*p* = 0.001). No significant differences were observed in operation procedures (*p* = 0.958) (Table 2). In comparisons of pathological features, lymph node metastasis was more frequent in Mt than in Wt (*p* = 0.033), and lymphatic invasion was slightly more frequent in Mt than in Wt (*p* = 0.077). However, no significant differences were observed in pathological stages or recurrent patterns between Wt and Mt (*p* = 0.337 and *p* = 0.280, respectively).

**Table 2:** Comparison of clinicopathological features between patients with Mt and Wt

Total *n* = 237	Mt (*n* = 108)	Wt (*n* = 129)	*P* values^a^
Age	66.5	66.1	0.791^b^
Male, (%)	48 (44.4)	85 (65.9)	0.001
Smoking history, (%)	52 (48.1)	94 (72.9)	< 0.001
Surgical procedure, (%)			
pneumonectomy	2 (1.9)	3 (2.3)	
lobectomy	104 (96.2)	124 (96.1)	
segmentectomy	2 (1.9)	2 (1.6)	0.958
Pathological tumor size, (mm)	33.9 (11–100)	40.0 (11–210)	0.019^b^
Pathological stage, (%)			
I	24 (22.2)	36 (27.9)	
II	26 (24.1)	36 (27.9)	
III	58 (53.7)	57 (44.2)	0.337
Lymphatic invasion, (%)	61 (56.4)	58 (45.0)	0.077
Vascular invasion, (%)	68 (63.0)	83 (64.3)	0.826
Pleural invasion, (%)	50 (46.3)	72 (55.8)	0.144
Nodal invasion, (%)	81 (75.0)	80 (62.0)	0.033
Recurrence pattern			
locoregional	29 (26.9)	43 (33.3)	
systemic	79 (73.1)	86 (66.7)	0.280
Administration of *EGFR* - TKI	81 (75.0)	7 (5.4)	< 0.001

^a^Fisher’s exact test

^b^Student’s *t*-test

Mt *EGFR* mutant, Wt *EGFR* wild-type, TKI tyrosine kinase inhibitor

**Fig. 1:**
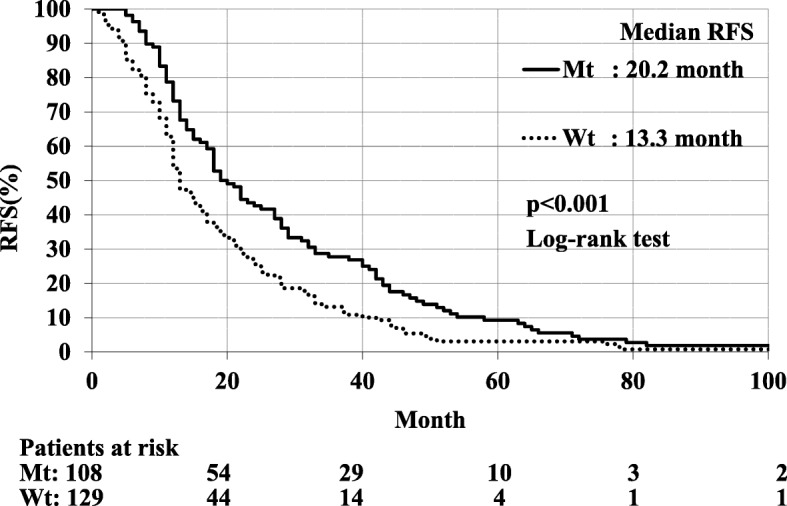
Median RFS was significantly better for lung adenocarcinoma patients with Mt than Wt; median RFS were 20.2 months and 13.3 months, respectively (*p* < 0.001)

**Fig. 2:**
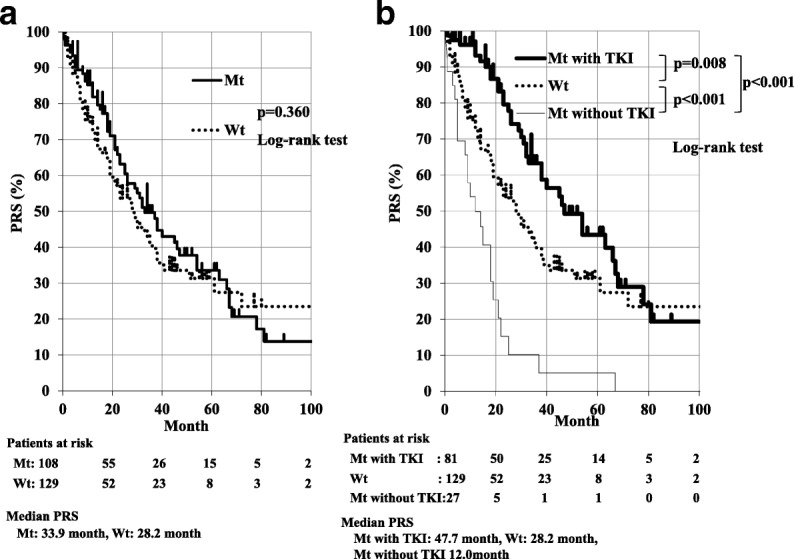
No significant differences were observed in median PRS between Mt and Wt; median PRS were 33.9 months and 28.2 months, respectively (*p* = 0.360, Fig. 2a). PRS was significantly better in Mt with *EGFR* - TKI than in Wt and Mt patients without *EGFR* - TKI (*p* = 0.008 and *p* < 0.001, respectively). PRS was also significantly better in Wt than in Mt patients without *EGFR* - TKI (*p* < 0.001, Fig. 2b)

RFS was significantly better in Mt than in Wt patients; median RFS for Mt and Wt patients were 20.2 months and 13.3 months, respectively (*p* < 0.001, Fig. 1). No significant differences were observed in PRS between Mt and Wt patients; median PRS for Mt and Wt patients were 33.9 months and 28.2 months, respectively (*p* = 0.360, Fig. 2a). As shown in Fig. 2b, PRS was significantly better in Mt with *EGFR* - TKI than in Wt and Mt patients without *EGFR* - TKI (*p* = 0.008 and *p* < 0.001, respectively). PRS was also significantly better in Wt than in Mt patients without *EGFR* - TKI (p < 0.001, Fig. 2b).

Univariate and multivariate analyses for RFS were shown in Table 3. In the univariate analysis, gender, smoking history, pathological T factor, lymphatic invasion, and the *EGFR* mutation status were identified as prognostic factors. In the multivariate analysis, *EGFR* mutations (hazard ratio [HR] = 0.68, 95% confidence interval [CI], 0.52–0.89, *p* = 0.005) and lymphatic invasion (HR = 1.34, 95% CI, 1.03–1.74, *p* = 0.029) were independent prognostic factors for RFS. Mt patients without lymphatic invasion had significantly better RFS than Mt patients with lymphatic invasion; median RFS were 29.0 (22.8–35.8) months and 15.9 (13.2–19.1) months, respectively (*p* = 0.020).

**Table 3:** Multivariate Cox’s Proportional Hazard Regression Model for RFS

Variable	Univariate analysis	Multivariate analysis		
	*p* value	HR	95% CI	*p* value
Age (> 65)	0.809			
Gender (Male)	< 0.001	1.09	0.71–1.68	0.687
Smoking history	< 0.001	1.39	0.91–2.13	0.125
Pathological T factor	< 0.001	1.15	0.94–1.42	0.172
Pathological N factor	0.353			
Pathological stage	0.119			
Vessel invasion	0.314			
Lymphatic invasion	0.027	1.34	1.03–1.74	0.029
Pleural invasion	0.231			
*EGFR* mutation (+/−)	< 0.001	0.68	0.52–0.89	0.005

*RFS* Relapse-free survival, *EGFR* epidermal growth factor receptor, *HR* Hazards ratio, *CI* Confidence interval

Univariate and multivariate analyses for PRS were shown in Table 4. In the univariate analysis, age, smoking history, pathological T factor, the administration of *EGFR* - TKI, and the recurrence interval were identified as prognostic factors for PRS, whereas the *EGFR* mutation status was not a prognostic factor for PRS. In the multivariable analysis, age (HR = 1.63, 95% CI, 1.11–2.38, *p* = 0.012) and the administration of *EGFR* - TKI (HR = 0.60, 95% CI, 0.40–0.89, p = 0.012) were independent prognostic factors.

**Table 4:** Multivariate Cox’s Proportional Hazard Regression Model for PRS

Variable	Univariate analysis	Multivariate analysis		
	*p* value	HR	95% CI	*p* value
Age (> 65)	0.014	1.63	1.11–2.38	0.012
Gender (Male)	0.178			
Smoking history	0.008	1.38	0.93–2.05	0.113
Pathological T factor	< 0.001	1.07	0.80–1.45	0.638
Pathological N factor	0.831			
Pathological stage	0.684			
Vessel invasion	0.722			
Lymphatic invasion	0.787			
Pleural invasion	0.659			
Systemic recurrence (vs. locoregional)	0.072			
*EGFR* mutation (+/−)	0.360			
Administration of *EGFR* - TKI	< 0.001	0.60	0.40–0.89	0.012
Recurrence interval (24 < vs 24≥)	0.017	1.35	0.91–2.01	0.142

*PRS* post-relapse survival, *EGFR* epidermal growth factor receptor, *TKI* tyrosine kinase inhibitor, *HR* Hazards ratio, *CI* Confidence interval

**Fig. 3:**
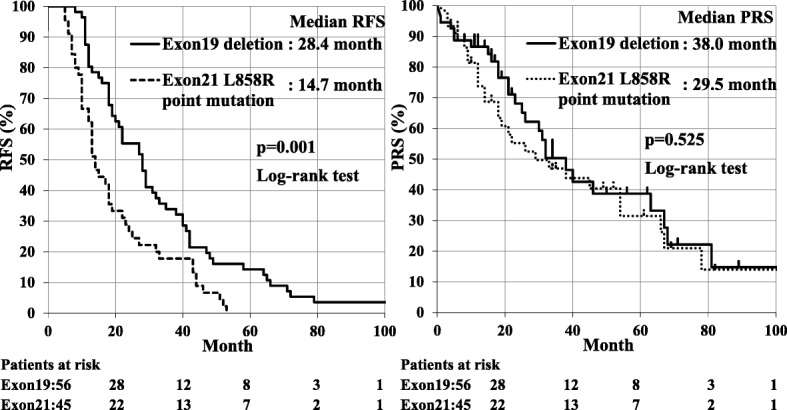
Median RFS was significantly poorer for lung cancer patients with the Exon 21 L858R point mutation (*n* = 45) than those with the Exon 19 deletion (*n* = 56); median RFS were 14.7 months and 28.4 months, respectively (*p* = 0.001). No significant differences were observed between the two *EGFR* mutations; median PRS were 29.5 months and 38.0 months, respectively (*p* = 0.525)

In Fig. 3, the prognosis of patients with *EGFR* exon 21 L858R point mutation (L858R) lung cancer (*n* = 45) and *EGFR* exon 19 deletion (19 Del) lung cancer were compared. Patients with L858R lung cancer had significantly poorer RFS than those with 19 Del lung cancer; median RFS were 14.7 months and 28.4 months, respectively (*p* = 0.001). No significant differences were observed in the frequency of using *EGFR* - TKI between patients with L858R and 19 Del lung cancer (68.9% vs 80.4%, respectively; *p* = 0.184). Moreover, there was no significant difference in PRS between patients with L858R and 19 Del lung cancer; median PRS were 29.5 months and 38.0 months, respectively (*p* = 0.525).

## Discussion

Mt patients had better RFS than Wt patients (20.2 vs. 13.3 months, *p* < 0.001), and Mt was an independent factor for favorable RFS in the present study (HR = 0.68, *p* = 0.005). These results imply that Mt tumors take a longer period to recur after curative surgery and exhibit less aggressive behavior than Wt tumors. No significant differences were observed in PRS; however, Mt patients had slightly better survival than Wt patients (33.9 vs. 28.2 months, *p* = 0.360). PRS was significantly better in the order of Mt with *EGFR* - TKI, Wt, and Mt without *EGFR* - TKI (Fig. 2b). Independent prognostic factors for PRS were *EGFR* - TKI and age, and the *EGFR* mutation status did not influence PRS. The *EGFR* mutation status did not independently affect the prognosis of patients with pulmonary adenocarcinoma after recurrence, and the administration of *EGFR* - TKI was mandatory for improving PRS in patients with recurrent Mt.

Previous studies reported that the prognosis of patients with Mt who underwent complete resection of the lung was better than those with Wt; however, the reasons for this difference were unclear [9–11]. In pathological examinations of adenocarcinoma of the lung, the lepidic growth pattern was more frequently observed in Mt than in Wt [6–9], and Mt was associated with adenocarcinoma in situ and minimally invasive adenocarcinoma, which rarely recur [9]. Since the prognosis of Mt may strongly depend on the frequency of adenocarcinoma in situ and minimally invasive adenocarcinoma of the lung, we intended to include recurrent adenocarcinoma of the lung in order to exclude these low-grade adenocarcinomas; none of the tumors in the present study were adenocarcinoma in situ or minimally invasive adenocarcinoma (data not shown) which is defined in WHO classification 2015 and consistent with low-grade adenocarcinoma. The period after curative surgery to recurrence was longer in Mt patients than in Wt patients, and this result implied that Mt tumors had a less aggressive growth nature than Wt tumors among recurrent adenocarcinomas of the lung.

Watanabe et al. previously reported the bimodal distribution of recurrence patterns after curative resection of adenocarcinoma of the lung; the predilection periods of pulmonary adenocarcinoma recurring after curative surgery were 6–14 months and 20–22 months [15]. In the present study, median RFS for Wt and Mt patients were 13.3 months and 20.2, respectively. This difference in RFS between Mt and Wt may result in the bimodal distribution of the recurrence pattern after curative resection for adenocarcinoma of the lung; the early recurrence of Wt and delayed recurrence of Mt. The *EGFR* mutation status provides thoracic surgeons with useful information on postoperative follow-up strategies for adenocarcinoma of the lung. Nearly 10% of recurrent Mt was observed more than 5 years after curative surgery in this study, and this result implies that patients with Mt need to be followed-up for a longer period than those with Wt.

Lymphatic invasion was another independent prognostic factor for RFS along with the *EGFR* mutation status. Median RFS for patients with Mt without lymphatic invasion was 29.0 (22.8–35.8) months and these tumors were considered to be less aggressive among Mt. Lymphatic invasion is associated with recurrence and has been identified as a poor prognostic factor for the overall survival of patients with early-stage lung cancer after surgery [16, 17]. In the present study, lymphatic invasion was not a prognostic factor for PRS in patients with recurrent adenocarcinoma of the lung. Lymphatic invasion only affected the RFS of patients with pulmonary adenocarcinoma after surgery.

According to randomized clinical trials on *EGFR* - TKI for unresectable advanced non-small-cell lung cancer, progression-free survival and overall survival were reported to be 9.2–11.0 months and 19.3–34.8 months, respectively [1–3]. Although large-scale randomized clinical trials on *EGFR* - TKI exclusively for recurrent Mt have yet to be conducted, several retrospective analyses reported that median PRS for recurrent Mt patients who received *EGFR* - TKI was 37.1–63.4 months [18–20]. Median PRS was 47.7 (33.9–67.3) months in this study, which was consistent with previous findings. Since *EGFR* - TKI were identified as a prognostic factor for favorable PRS in this study, a long-term follow-up is considered mandatory for patients with Mt in order to ensure that they receive *EGFR* - TKI therapy.

Patients with 19 Del lung cancer had better RFS than those with L858R lung cancer in the present study (Fig. 3, *p* = 0.001), and this result implied that 19 Del lung cancer exhibits less aggressive behavior than L858R lung cancer among recurrent pulmonary adenocarcinoma. We previously reported that disease-free survival was better for patients with pN1-pN2 19 Del lung cancer than those with pN1-pN2 L858R lung cancer (38.8% vs. 11.8%, p = 0.001), and overall survival was slightly better in patients with pN1-pN2 19 Del lung cancer than in those with pN1-pN2 L858R lung cancer (78.3% vs. 48.3%, *p* = 0.123) [12]. Another study reported that 19 Del lung cancer had better disease-free survival and overall survival than L858R lung cancer among stage III lung cancers after resection of the lung [21]. The postoperative prognosis of Mt patients might differ according to the major *EGFR* mutation among resectable advanced and recurrent adenocarcinomas of the lung. However, recent study from Takamochi reported that RFS did not differ for patients with L858R lung cancer and 19 Del lung cancer [22]. Further analysis in larger cohort was necessary in order to clarify differences between the two major *EGFR* mutations.

Among unresectable advanced lung cancers, previous studies reported better responses to *EGFR* - TKI, progression-free survival, and overall survival in patients with 19 Del lung cancer than in those with L858R lung cancer [23–26]. Although the specific reasons for these differences were unclear, biomolecular studies suggested that the more favorable prognosis of patients with 19 Del lung cancer was due to better responses to *EGFR* - TKI by 19 Del lung cancer than by L858R lung cancer [27, 28]. In contrast to previous findings, no significant differences were observed in PRS between patients with 19 Del and L858R lung cancer, although patients with 19 Del had slightly longer PRS than those with L858R (38.0 months and 29.5 months, respectively; *p* = 0.525). Although the reasons for the discordance between previous findings and the present results are unclear, the following three reasons have been suggested. Patients with recurrent lung cancer were included exclusively in this study. This study was based on a small number. *EGFR* - TKI were not administered to all patients with Mt.

There were some limitations in the present study. First, since this study was a retrospective analysis, there was a possible selection bias of Mt and Wt patients. Second, PRS was slightly better in Mt than in Wt (approximately 6 months) but not statistically significant. This study may have been underpowered due to the small sample size. Third, lung cancers harboring minor *EGFR* mutations, which are considered to be refractory to *EGFR* - TKI, were included in Mt, whereas lung cancers harboring anaplastic lymphoma kinase genes or ROS-1 gene mutations were included in Wt. Since the population of Mt and Wt was considered to be heterogeneous, further analyses on prognosis based on each gene mutation are considered to be necessary for analyzing the characteristics of each adenocarcinoma of the lung. Fourth, in the present study, all patients had recurrent lung cancer, and, thus, further studies are needed in order to examine predictive factors that explain the recurrence of adenocarcinoma of the lung after curative surgery.

## Conclusions

Mt takes a longer period to recur after curative surgery than Wt, and Mt was considered to exhibit less aggressive behavior than Wt. The *EGFR* mutation status may predict not only responsiveness to *EGFR* - TKI, but also the period to recurrence after the resection of each pulmonary adenocarcinoma. The longer follow-up of patients with Mt beyond 5 years is considered necessary and *EGFR* - TKI need to be administered to patients with Mt after recurrence.

## Abbreviations

19 Del: EGFR exon 19 deletion
CI: confidence interval
CT: computed tomography
EGFR: epidermal growth factor receptor
HR: hazard ratio
L858R: EGFR exon 21 L858R point mutation
Mt: EGFR mutant adenocarcinoma of the lung
PRS: post-relapse survival
RFS: relapse-free survival
TKI: tyrosine kinase inhibitors
Wt: EGFR wild-type adenocarcinoma of the lung
